# Medication and supplement use for managing joint symptoms among patients with knee and hip osteoarthritis: a cross-sectional study

**DOI:** 10.1186/1471-2474-13-47

**Published:** 2012-03-29

**Authors:** Jeffrey B Driban, Sara A Boehret, Easwaran Balasubramanian, Nicole M Cattano, Joseph Glutting, Michael R Sitler

**Affiliations:** 1 Division of Rheumatology, Tufts Medical Center, 800 Washington Street, Box #406, Boston, MA 02111, USA; 2 Department of Kinesiology, Temple University, 1800 N. Broad Street, Philadelphia, PA 19122, USA; 3 Department of Orthopaedic Surgery and Sports Medicine, Temple University, 3509 N. Broad Street, 5th Floor Boyer Pavilion, Philadelphia, PA 19140, USA; 4 Department of Sports Medicine, West Chester University of Pennsylvania, 855 S. New Street, 313 Sturzebecker HSC, West Chester, PA 19383, USA; 5 School of Education, 221 Willard Hall, University of Delaware, Newark, DE 19716, USA; 6 College of Health Professions and Social Work, Temple University, 3307 N. Broad Street, Suite 300, Philadelphia, PA 19140, USA

## Abstract

**Background:**

The purpose was to determine the professionally-guided and self-guided medication and supplement use for joint symptom management among patients with knee and/or hip osteoarthritis (OA) in an urban hospital-based outpatient orthopedic practice.

**Methods:**

The study design was cross-sectional. Patients diagnosed by radiographs and clinical symptoms with knee and/or hip OA were recruited from an inner-city hospital-based outpatient orthopaedic office. A total of 184 patients were queried for their participation. Four investigator-generated, interview-based questionnaires were used. Sampling error was ± 7.84%. Logistic regression models and Fisher Exact Tests were performed to determine factors that may be associated with negative behaviors related to medication or supplement use (e.g., reporting medication as ineffective, using multiple medications in the same day to manage symptoms). Odds ratios (OR) and 95% confidence intervals (CI) were calculated for significant findings.

**Results:**

Among the 162 participants, a majority reported professionally-guided recommendations and over 40% reported at least one self-guided intervention. 37 participants reported dual-use during the same day, and among those,15 reported dual-use at the same time. Among participants taking multiple interventions in the same day, 40.5% reported using prescription and over-the-counter medications. Use of multiple medications or supplements in one day was more common among participants who reported OA at multiple joints (OR [95% CI] = 2.48 [1.03 to 5.96]) but less common among participants who did not complete high school (OR [95% CI] = 0.26 [0.08 to 0.83]). Of the 15 participants who reported dual-use at the same time, 11 were professionally-guided, 5 were professional and self-guided, and 1 was solely self-guided. Overall, 28% of participants reported their intervention as ineffective, sought an alternative method to achieve symptomatic relief, or were prescribed a stronger medication. Participants who reported not always taking their medication consistently for 2 weeks were more likely to report their medication as ineffective (OR [95% CI] = 2.87 [1.19 to 6.92]).

**Conclusions:**

Both professional and self-guided medications and supplements are used by inner city OA patients to manage their joint symptoms. It is important for clinicians to discuss with these patients how to effectively manage multiple joint symptoms, the importance of taking medications as prescribed, and what they should if they believe a treatment is ineffective or their medication runs out.

## Background

Osteoarthritis (OA), the most common form of arthritis, is a heterogeneous disease characterized by multi-tissue failure of the synovial joints and an illness defined by patient reported symptoms (e.g., pain, stiffness) [1]. Symptom management, is important to an individual's quality of life and function, and is the primary focus of current OA treatment [2]. OA symptom management includes both professionally-guided as well as self-guided care interventions [3,4]. Self-guided care is undertaken based on information obtained from professionals and other personal experiences [3] and has long been recognized for its importance in chronic disease management [5]. Self-guided care for OA includes over-the-counter (OTC) medications, prescription drugs not used for their prescribed purpose, and dietary supplements that individuals take to ameliorate their symptoms [3]. Professionally-guided care typically consists of prescription and OTC medications, injections, and physical therapy prescribed by physicians [3].

In the United States, it is not fully understood how OA patients manage their symptoms with professionally-guided and self-guided pharmacological care strategies. Among patients taking a non-steroidal anti-inflammatory drug (NSAID) for management of their joint discomfort (e.g., rheumatoid arthritis, OA, low back pain), approximately 26% also use other analgesic medications [6]. Specifically among OA patients, it is estimated that 70% take a prescription medication and 44 to 70% take an OTC analgesic to control joint symptoms [3]. While a high percentage of OA patients take prescription and/or OTC analgesics, it is unclear how many OA patients combine medications. In addition, the pattern of use within the same day and at the same time is unknown. As the incidence of OA increases, the use of multiple medications to control for symptoms is likely to increase as well.

In spite of the guidelines and recommendations that exist for the management of OA, inappropriate multiple analgesic medication use among OA patients may represent an additional burden on a health-care system that is struggling to adapt to an increasing prevalence of chronic diseases [7]. In the United Kingdom and United States, 25 and 54% of patients, respectively, are not aware of potential drug side effects and do not understand that analgesics can be taken prophylactically or at the onset of pain [6,8]. Twenty-six and 8% of patients over medicate with OTC and prescription NSAIDs, respectively. In the United States it has not been determined which medications are being combined among OA patients and if these patients are taking the medications at the same time, potentially placing their health at risk (e.g., gastrointestinal toxicity, hepatic complications, renal complications) [6,8]. The purpose of our study was to determine the professionally-guided and self-guided pharmacological and supplement use for joint symptom management among knee and hip OA patients in an urban hospital-based outpatient orthopedic practice. The outcome of this study is intended to improve clinical care and education of OA patients.

## Methods

### Participants

Participants were recruited from Temple University Hospital's Department of Orthopedics inner-city outpatient office between December 2009 and March 2011. The participants were all under the care of one of 3 board-certified orthopedic physicians for knee and/or hip OA. The OA diagnosis was based on x-ray (Kellgren-Lawrence score ≥ 1) [9] as well as knee or hip clinical symptoms (e.g., pain, stiffness, swelling, impaired function). Exclusion criteria were any other rheumatic disease (e.g., rheumatoid arthritis, lupus, gout) or diagnosis of complex regional pain syndrome. A total of 184 patients were queried for their participation in the study of which 22 patients were excluded and/or declined to participate. Characteristics of those not willing to participate are unknown. Institutional Review Board approval by Temple University's Institutional Review Board as well as written informed consent were obtained prior to data collection.

### Research design

The study design was cross-sectional. Four interview-based questionnaires were used to address the specific aims and research questions of the study: Demographic and Health History Questionnaire, Health History Follow-up Questionnaire, Pharmacological and Supplement Use Assessment Questionnaire, and Pharmacological and Supplement Use Follow-up Questionnaire (see Additional file 1). A priority of 8 specific aims with several specific research questions were identified and approached as an exploratory analysis in this study. Professionally-guided care was defined as a prescription medication, OTC medication, or dietary supplement recommended by a physician. Self-guided care was defined as any OTC medication, prescription drug not used for its original purpose, or dietary supplement taken without the recommendation from a physician.

### Instrumentation

Four interview-based questionnaires were used to obtain information about the participant's health history and pharmacological and supplemental use: two initial questionnaires and two follow-up questionnaires. The questionnaires were developed by the research team and reviewed by two statisticians: one specializing in psychoeducational assessment research and the other in community-based epidemiology. All of the questionnaires were interview based. The questionnaires were an item-by-item design, where each question could be scored individually. The interviewer practiced the procedures and data coding on 18 non-study patients with knee OA prior to starting the study.

#### Demographic and health history questionnaire

The Demographic and Health History Questionnaire was used to obtain participant general health as well as knee and/or hip joint specific information. It consisted of participant identity information and questions concerning his/her demographics, education level, physical activity level, knee and/or hip injury and treatments (e.g., medication use), and general medical history. In addition, each participant was asked to list all medications he/she was currently taking. The Demographic and Health History Questionnaire consisted of "yes" or "no", Likert-type 5-point scale, as well as open- and close-ended questions.

#### Health history follow-up questionnaire

The Health History Follow-up Questionnaire was used to discern additional information in follow-up to the Demographic and Health History Questionnaire. Follow-up questions were prompted if a participant responded "yes" to any questions regarding previous injury, surgery, other arthritic joint, other treatments used for OA, and sport participation history.

#### Pharmacological and supplement use assessment questionnaire

The Pharmacological and Supplement Use Assessment Questionnaire was used to identify the specific medications commonly used by the participant as part of his/her knee and/or hip OA joint symptom management. Drug categories were: NSAIDs, herbal remedies, nutritional supplements, injections, acetaminophen, pain relieving medications, and any other drugs for joints. In addition, each participant provided information about when he/she had used each drug: in the last 24 hours, greater than 24 hours but less than 2 weeks ago, 2 to less than 4 weeks ago, 4 weeks to less than 3 months ago, or more than 3 months ago.

#### Pharmacological and supplement use follow-up questionnaire

The Pharmacological and Supplement Use Follow-up Questionnaire was used to discern additional details concerning the participant's specific medication use. Follow-up questions addressed the drug dose; OTC versus prescription drug; if the drug was taken as directed and consistently; if more than one drug was being taken; and if the drug was no longer being taken, and, if not, why it was stopped.

### Procedures

Participants were recruited from Temple University Hospital's Department of Orthopedics and informed of the study's purpose. Those who agreed to participate read and signed the study compliance forms. Next, the participant was interviewed by one of the coauthors (SB) using the Demographic and Health History Questionnaire as well as Pharmacological and Supplement Use Assessment Questionnaire in sequential order in a private examination room. The interview had no time limit, but the average interview took between 15 and 20 minutes to complete. Based on the responses to each of the initial questionnaires, participants were then interviewed by the same co-author using the appropriate follow-up questionnaire(s).

### Statistical analysis

Data analyses included descriptive statistics using the SAS 9.2 (SAS Institute, Cary, NC) statistical package. Items were analyzed with mean, standard deviation, frequency and/or percentage statistics. Sampling error was ± 7.84%.

Logistic regressions were performed to predict 1) same day dual-medication use, 2) participants that only used self-guided recommendations compared to those that only used professionally-guided recommendations, 3) use of medication as directed, and 4) reporting a medication as ineffective or requiring a stronger medication. Potential predictors were ethnicity, sex, obesity, age, reporting multiple OA joints, and education. Models were calculated with SPSS 20 (IBM Corporation, Armonk, NY) and considered significant if the overall p-value was ≤ 0.05. The sensitivity and specificity of significant models was calculated with Diagnostic Utility Statistics [10].

Two Fisher's Exact Tests were performed in SAS 9.2 to further assess potential confounders for patients reporting their medication as ineffective or requiring a stronger medication. One test assessed the association between participants reporting their medication as ineffective and not always using medication as directed. The other test evaluated the association between participants reporting their medication as ineffective and not always using medication consistently. Fishers exact tests were considered significant if the p-value was ≤ 0.05.

## Results

Of the 162 participants, 151 participants had primary complaints of knee OA (53% [n = 80] self-reported OA in other joints), and 11 had hip OA (82% [n = 9] self-reported OA in other joints). Kellgren-Lawrence scores were distributed as follows: 1 (1.23%), 2 (21.60%), 3 (41.36%), and 4 (35.80%). Additional descriptive information is provided in Table 1. Furthermore, Table 2 includes the overall number of reported medications and supplements. Five participants reported not taking any medications or supplements. Three of the five participants were managing their joint symptoms with intra-articular injections only. Another participant that reported no medications or supplements stated that she was avoiding medication use because of an adverse event with an intra-articular injection. The final participant reported no medication or supplement use but did not provide a reason for doing so. These 5 participants were excluded from the remainder of the analyses.

**Table 1:** Study sample characteristics (*n *= 162)

Variable	Distribution *n *(%) or *m *± *sd*
Female	108 (66.7%)

Ethnicity	

African descent	118 (72.8%)

Caucasian	28 (17.3%)

Hispanic and/or Other	16 (9.9%)

Education	

Less than High School	44 (27.2%)

High School (or Equivalent)	78 (48.2%)

Beyond High School	40 (24.7%)

Age (years)	59 ± 10

Body mass index (kg/m^2^)	35.1 ± 7.6

m = mean, sd = standard deviation.

**Table 2:** Pharmacological and supplemental use among osteoarthritis knee and hip patients

Type, source and pattern of use	Frequency	Percentage
Overall number of reported interventions*		

Zero	5	3.1

One	92	56.8

Two	55	34.0

Three or More	10	6.2

Class of intervention‡		

NSAID	106	65.4

Other analgesics	55	34.0

Acetaminophen	38	23.5

Nutritional supplement	11	06.8

Level of intervention‡		

Rx user only	80	49.4

OTC user only	46	28.4

Rx and OTC user	31	19.1

Source of recommendation‡		

PCP only	41	25.3

Self-guided only	39	24.1

Orthopedic only	28	17.3

PCP and self-guided	16	09.9

Multiple professional sources	12	07.4

Orthopaedic and self-guided	11	06.8

Other sources	10	06.2

Recall medication dose‡		

All	76	46.9

None	53	32.7

Some	28	17.3

Medications used as directed‡		

Always	110	67.9

Never	34	21.0

Sometimes	13	08.0

2 week consistent medication use‡		

Always	88	54.3

Never	48	29.6

Sometimes	21	13.0

* Interventions = prescription or over-the-counter medication as well as supplements.

‡ N = 157/162 participants whom reported using a pharmacological intervention, 5 participants reported using none.

NSAID = non-steroidal anti-inflammatory drug, Rx = prescription, OTC = over-the-counter, and PCP = primary care physician.

The type and source of pharmacological and supplement use information is reported in Table 2. The most common class of intervention was NSAIDs (particularly naproxen [29.6%] and ibuprofen [24.1%]) followed by other analgesics (i.e., prescription analgesics), acetaminophen, and nutritional supplements. Half of the participants reported using prescription medications only, while approximately a quarter of the participants used OTC medications and supplements only. Almost 20% of the participants used both prescription and OTC medications and supplements. Most patients (55.6%) reported only professionally-guided recommendations, 24.1% indicated only self-guided recommendations, and 17.3% reported professionally and self-guided recommendations for their medication and supplement use. Results of the logistic regression used to evaluate predictors (i.e., ethnicity, sex, obesity, age, reporting multiple OA joints, and education) of a participant only using self-guided recommendations compared to only using professionally-guided recommendations was not significant (*p *= 0.149).

Also reported in Table 2 is the pattern of medication use information. Less than half of the participants were always able to recall their medication dose, while the balance was either unable or partially able to do so. Twenty-nine percent of the participants reported never or only sometimes using their medication and/or supplement as directed, while the balance reported they always took their medication and/or supplement as directed. Results of the logistic regression used to evaluate predictors (i.e., ethnicity, sex, obesity, age, reporting multiple OA joints, and education) of a participant not always using their medication as directed was not significant (*p *= 0.139). Approximately half of the participants reported taking their medication and/or supplement consistently for 2 weeks whereas the others did not.

Thirty-seven (23%) participants were same day dual-medication and/or supplement users while 15 (9%) participants were taking the combinations at the same time. Dual-medication and/or supplement use pattern information is represented in Table 3. The majority of participants used multiple classes of medications at the same time. Eighty-six percent of dual-use participants reported using prescription medications only or both prescription and OTC medications at the same time.

**Table 3:** Day and time patterns of multiple-medication use among osteoarthritis knee and hip patients

Multiple-medication use	**Same day (n = 37)**		**Same time (n = 15)**	
	**Frequency**	**Percentage**	**Frequency**	**Percentage**

Class of intervention				

Multiple-classes	30	81.1	14	93.3

NSAID only	5	13.5	0	0

Other	2	05.4	1	06.7

Level of intervention				

Rx and OTC user	15	40.5	5	33.3

Rx user only	13	35.1	8	53.3

OTC user only	9	24.3	2	13.3

NSAID = non-steroidal anti-inflammatory drug, Rx = prescription, and OTC = over-the-counter.

Results of the logistic regression to evaluate predictors of an individual being a same day dual-medication user were statistically significant. The Homer-Lemeshow goodness-of-fit test showed a good model fit with the data (*p *= 0.912). The overall accuracy of the model was 75.8% with a sensitivity of 44.4% and specificity of 77.7%. Participants reporting OA at multiple joints were more likely to be same day dual-medication users (odds ratio [95% confidence interval; CI] = 2.48 [1.03 to 5.96]) after controlling for covariates (i.e., ethnicity, sex, obesity, age, and education). In contrast, participants with less than a high school education were 3.82 times less likely to be same day dual-medication users (odds ratio [95% CI] = 0.26 [0.08 to 0.83]) than participants with a high school education or greater.

The source of recommendation of same time dual-medication and/or supplement use is reported in Table 4. Two participants reported 2 distinct patterns of dual use, resulting in fifteen participants reporting 17 dual-use combinations. Of these 17 dual-use combinations, 11 (65%) were professionally-guided based recommendations (physician-physician), 5 (29%) were professional and self-guided-based recommendations (physician-self), and 1 (6%) was a self-guided-based recommendation (self-self). Common combinations included NSAIDs plus analgesics (e.g., acetaminophen, tramadol; 9 out of 17) and medication plus supplement (e.g., calcium glucosamine; 5 out of 17).

**Table 4:** Source of recommendation of dual-medication or nutritional supplement use among osteoarthritis knee and hip patients

Participant #	Drug 1	Drug 2	Source of Recommendation	
			
			Drug 1	Drug 2
1	Indomethacin	Acetaminophen	PCP	Self-guided

2	Diclofenac	Tramadol	Orthopedic	Orthopedic

3	Acetaminophen	Calcium	Orthopedic	Self-guided

4	Ibuprofen	Calcium	Self-guided	Self-guided

4	Naproxen	Calcium	PCP	Self-guided

5	Naproxen	Glucosamine	PCP	PCP

6	Naproxen	Acetaminophen + Codeine	PCP	PCP

7	Ibuprofen	Acetaminophen + Oxycodone	Self-guided	PCP

8	Tramadol	Acetaminophen + Codeine	Orthopedic	PCP

9	Naproxen	Acetaminophen	PCP	PCP

10	Oxycodone	Hydromorphone	Psychiatrist	Psychiatrist

11	Diclofenac	Tramadol	Orthopedic	Orthopedic

12	Meloxicam	Tramadol	Orthopedic	Orthopedic

12	Ibuprofen	Tramadol	Self-guided	Orthopedic

13	Glucosamine	Chondroitin	Orthopedic	Orthopedic

14	Ibuprofen	Acetaminophen + Oxycodone	PCP	ER

15	Tramadol	Acetaminophen + Oxycodone	PCP	PMC

Participants 4 and 12 had 2 distinct patterns of use.

PCP = primary care physician, Orthopedic = orthopedic surgeon, ER = emergency room physician, and PMC = pain management center.

Table 5 describes several additional behaviors that participants reported to manage joint symptoms. Twenty-nine (18.5%) participants reported their medication as ineffective, required a stronger medication, or used illegal drugs to manage their symptoms. Alternatively, 15 (9.6%) participants sought an alternative method to achieve symptom relief (i.e., emergency room for symptom management, used other person's prescription medication, or used medication prescribed for an alternative ailment). Overall, 28% of participants reported their intervention as ineffective, sought an alternative method to achieve symptom relief, or were prescribed a stronger medication. Results of the logistic regression used to evaluate predictors (i.e., ethnicity, sex, obesity, age, reporting multiple OA joints, and education) of a participant reporting their medication as ineffective or requiring a stronger medication was not significant (*p *= 0.087). Conversely, Fisher's Exact Test revealed that participants who reported not always taking their medication consistently for 2 weeks were more likely to report their medication as ineffective (24.6% of participants) compared to participants always taking their medication consistently for 2 weeks (10.2% of participants; odds ratio [95% CI] = 2.87 [1.19 to 6.92], *p *= 0.010). No association was found between participants reporting medication as ineffective or requiring stronger medication and not always using medication as directed (*p *= 0.153). Thirteen participants reported other reasons for discontinuing their medication (e.g., no longer needed the medication, physician concerns regarding potential risks, patient fear of addiction, insurance refused to cover medication).

**Table 5:** Additional behaviors among medication and supplement users (*n *= 157)

Behavior	Distribution *n *(%)
Reported Medication/Supplement As Ineffective or were Prescribed Stronger Dose	26 (16.6%)

Use of Illegal Drugs (e.g., marijuana)	3 (1.9%)

Use of Other Person's Prescription Medication	3 (1.9%)

Use of Medication Prescribed for Other Ailments	7 (4.5%)

Received Medication from Emergency Room	5 (3.2%)

Adverse Events from Medications/Supplements	5 (3.2%)

Stopped Medication Because the Prescription Ran out	17 (10.8%)

Stopped Medication for Miscellaneous Reasons	13 (8.3%)

m = mean, sd = standard deviation.

## Discussion

Although a majority of participants reported using professionally-guided recommendations, over 40% reported using at least one self-guided pharmacological and/or supplement intervention to manage their knee OA joint symptoms. Of the 15 participants who reported dual-use at the same time, 11 were professionally-guided, 5 were professional and self-guided, and 1 was solely self-guided based recommendation. Even with the combinations of professionally-guided, self-guided, and multiple sources of recommendation almost 17% of participants reported their intervention as ineffective or were prescribed a stronger medication. The only variable associated with participants reporting their intervention as ineffective (or requiring stronger medication) was not always taking their medication consistently for 2 weeks.

Our study revealed that nearly 20% of participants tried prescription and OTC medications to manage their joint symptoms. Among patients taking multiple interventions in the same day, 40.5% reported using prescription and OTC interventions. In Canada, 41% of patients recruited from urban primary care practices (n = 78 out of 190) reported using prescription medication and self-care products to manage their joint symptoms during one week [4]. It could not be determined if the Canadian patients were taking their medication combinations on the same day but this is likely since patients were asked to recall their medication use only for the week prior to the survey [4].

The overall combination of prescription and OTC interventions in our study may be lower than in the Canadian study [4] because they had fewer participants consuming only prescription medications (11% Canada versus 49% current study). The use of only OTC interventions was similar between studies (33% Canada versus 28% current study). The high prevalence of prescription medication use in the current study is similar to a cohort study conducted in Pittsburgh, Pennsylvania that had a high prevalence of prescription (54%) and OTC (63%) medication use during a 30-day period [3]. This study did not indicate whether participants were patients were taking a combination of prescriptions and OTC medications and supplements were included as a different class of interventions. Participants in the Pittsburgh study were requested to "bring in" their medications to be recorded on a medication inventory form. Based on these data, the authors reported that 70% of the participants were consuming prescription medications for OA symptom management. This is similar to our overall prescription medication use (69%) which was based on an interview-questionnaire format.

In the Pittsburgh study [3], among OTC medications, NSAIDs (58%: including COX-2 selective inhibitors) were the most commonly used drug class followed by other analgesics (55.7%). Our findings, although not limited to just OTC medications, were similar with NSAIDs being the most commonly used drug class utilized by 65% of participants followed by other analgesics (57%). These findings contradict current guidelines and recommendations that suggest acetaminophen should be the first line of defense over NSAIDs [2,11]. However, it is not known if the participants in our study had previously failed with acetaminophen treatment and therefore progressed to NSAIDs. Future research should include assessments of whether NSAIDs are commonly recommended after failure with other analgesics.

Approximately 23% of participants reported dual-use in the same day, while 9% of participants reported dual-use at the same time. While these data include OTC and prescription medications, the findings are similar to those reported by Albert et al [3] in which 23% of the patients used prescription medications in 2 or more classes. The extent to which the dual medication use occurred within the same day is not reported. Participants were more likely to use multiple medications on the same day if they reported that more than one joint had OA or if they had a high school education or greater. Future research should explore if patients are taking more than one medication because they believe each medication is for a particular joint or if one medication is only helpful for certain joints and not others. Although dual-use is not necessarily bad or against current guidelines and recommendations, four of five organizations currently offer no statement on dual-use [2,12-14]. The American College of Rheumatology is the only organization to offer a statement advocating dual-use for the treatment of OA [11]. While future research should evaluate medication dosage by using medication inventory forms, the data from our study indicates that the same-time dual-use combinations tend to be low-risk combinations (e.g., medications with supplements, NSAIDs with analgesics); however, recent research has raised concerns about the safety of combining NSAIDs with acetaminophen [15].

The current study identified several behaviors that have not been well studied in past OA research and may warrant further investigation. Thirteen (8%) participants reported using alternative methods to achieve symptomatic relief (e.g., misusing a prescription medication or consuming marijuana). Similarly, 26 participants reported stopping a medication because it was ineffective or they were prescribed a stronger medication. Based on these two findings it may be hypothesized that almost 1 in 5 patients in this cohort perceived one of their interventions to be ineffective. Participants that reported not taking medication consistently for 2 weeks were more likely to report their medication as ineffective. While longitudinal studies may be required to infer causality, it may be beneficial to suggest to patients that they should try new medications consistently (as prescribed) for at least 2 weeks. An additional 17 participants stopped taking their intervention because their prescription "ran out". We hypothesize that the reasons for this may have been due to socioeconomic status, access to healthcare, or effectiveness; but are yet to be determined as this was not the focus of the current study. These results highlight the need for clinicians to discuss with patients their satisfaction with interventions and what they should do if they believe a treatment is ineffective or their medication runs out.

The current study had several limitations. First was the sample size, which resulted in a ± 7.84% error rate. Another limitation of the study was that all participants were recruited from one inner-city orthopedic practice, narrowing the external validity of the study. The study also relied solely on self-reporting of medication use, which leaves the potential for misclassification of pharmacological and supplemental interventions. The Pharmacological and Supplement Use Assessment Questionnaire used in this study may have reduced the risk of misclassification because it provided a list of common NSAIDs and other interventions. Self-reported medication use was selected because of limitations within the current health system, a desire to capture over-the-counter medication use, and a preference to include any patient entering the clinic compared to a potentially biased sample of people that were willing to return with their medication bottles. We hypothesize that self-reporting was more likely to bias the results towards less medication use and safer patterns of medication use. Ostensibly, our data may represent a conservative reflection of how patients in an inner-city orthopaedic clinic manage their symptoms. Future larger, multi-center, epidemiology studies should be pursued. Although inclusion of medication inventory forms may help gather more information about current medication uses, the current instruments helped evaluate the behavior and influences on decisions concerning patient medication use. More research is needed to evaluate patient motives and alternative treatment strategies pursued through nontraditional mechanisms.

## Conclusions

In conclusion, in a small cohort of inner-city orthopaedic patients trying to manage their joint symptoms a large percent of patients initiated self-guided care, used OTC medications, were unable to recall their medication dose, and reported not taking their medication as directed. Furthermore, participants who reported never taking their medication consistently for 2 weeks were more likely to report their medication as ineffective. Some patients stopped taking their medications because their prescription ran out, sought additional symptomatic relief by taking multiple medications at the same time, and reported inappropriate behaviors to achieve symptomatic relief. Almost one in four patients reported taking multiple medications or supplements during a day to manage their symptoms. Patients reporting OA at multiple joints and a higher education were more likely to consume multiple interventions during a day to manage their symptoms. Fortunately, combinations of multiple medications at the same time appear to be low risk (e.g., acetaminophen with NSAIDs). Based on this study, it may be important for clinicians to discuss how to manage multiple joint symptoms, the importance of taking a medication as prescribed, and what they should do if they believe a treatment is ineffective or their medication runs out.

## Competing interests

The authors declare that they have no competing interests.

## Authors' contributions

JBD contributed to the conception and design, collection and assembly of data, analysis and interpretation of data, drafting/revisions of article, as well as final approval of the article. SAB contributed to the conception and design, collection and assembly of data, analysis and interpretation of data, drafting/revisions of article, as well as final approval of the article. EB contributed to the conception and design, collection and assembly of data, analysis and interpretation of data, drafting/revisions of article, as well as final approval of the article. NMC contributed to the conception and design, collection and assembly of data, analysis and interpretation of data, drafting/revisions of article, as well as final approval of the article. JG contributed to the conception and design, assembly of data, analysis and interpretation of data, drafting/revisions of article, as well as final approval of the article. MS contributed to the conception and design, collection and assembly of data, analysis and interpretation of data, drafting/revisions of article, as well as final approval of the article. All authors read and approved the final manuscript.

## Pre-publication history

The pre-publication history for this paper can be accessed here:

http://www.biomedcentral.com/1471-2474/13/47/prepub

## Supplementary Material

Additional file 1 **Demographic and Health History Questionnaire**. Click here for file
