# Supplementary material for: Medication and supplement use for managing joint symptoms among patients with knee and hip osteoarthritis: a cross-sectional study
Source: BMC Musculoskelet Disord. 2012 Mar 29;13:47. doi: 10.1186/1471-2474-13-47 (PMC3337291; doi:10.1186/1471-2474-13-47)
Supplement: Additional file 1 — Demographic and Health History Questionnaire. [file 1471-2474-13-47-S1.PDF]

Demographic and Health History Questionnaire

I. Patient Identifiers (by researcher)

Participant Number: _________ KL Score: ________ Date: ____________

Date of Birth: ______________ Height: ___________ Weight: ___________

Gender (Circle One): Male Female Knee/Hip OA(Circle One): Right Left Both

II. Demographic Information (by participant)

Please check the box that indicates your highest level of education:

☐ Less than High School ☐ High School + (but no college degree)

☐ High School or Equivalent ☐ Bachelor's Degree +

Please indicate your ethnicity (check as many as applies):

☐ Caucasian ☐ African ☐ Hispanic ☐ Asian ☐ Other: __________________

IV. Knee/Hip Injury and Treatment (by participant)

	YES	NO
1. Have you ever injured either knee/hip?<br>If yes, which knee/hip (circle one): Right Left Both	☐	☐
2. Have you ever had knee/hip surgery?<br>If yes, which knee/hip (circle one): Right Left Both	☐	☐
3. Does either knee/hip swell?<br>If yes, which knee/hip (circle one): Right Left Both<br>If yes, when was the last time (circle one)?<br>Less than 3 months ago More than 3 months ago	☐	☐
Did the swelling occur because of an injury?	☐	☐
Did the swelling require medical attention?	☐	☐
If your knee/hip is swollen now, how long has it been swollen (circle one):<br>Less than 1 week More than 1 week		
4. Have you been diagnosed with arthritis in a joint besides your knees/hips?	☐	☐
5. Do you walk with a limp?	☐	☐
6. Do you use a cane, walker, crutches or some other aid to walk?	☐	☐
7. Have you taken an anti-inflammatory medicine in the past three months?	☐	☐
8. Have you had an injection into your knee/hip within the last month?	☐	☐
9. Are you currently taking an anticoagulant (e.g., Warfarin or Coumadin)?	☐	☐
10. Have you taken an antibiotic within the last month?	☐	☐
11. Have you ever received any treatments for joint pain or arthritis?	☐	☐

Please circle on the following scale, the number that corresponds with the amount of difficulty you experienced during the following activities within the last 24 hours:

	NO DIFFICULTY				EXTREME DIFFICULTY
Daily Activities	0	1	2	3	4
Walking	0	1	2	3	4
On Stairs	0	1	2	3	4

Please circle on the following scale, the number that corresponds with the amount of pain you experienced during the following activities within the last 24 hours:

	NO PAIN				WORST PAIN IMAGINABLE
At Rest	0	1	2	3	4
Walking	0	1	2	3	4
On Stairs	0	1	2	3	4

IV. Physical Activity Level (by participant)

	YES	NO
1. Do you currently participate in some form of regular physical activity?	☐	☐
If yes, how many days per week? ___ How many minutes per day?___		
2. Have you ever played sport(s)?	☐	☐
If yes, what sport(s)?____________________________________		

V. General Medical Conditions (by participant)

Please indicate if you have ever been diagnosed by a physician with any of the following conditions.

	YES	NO	NOT SURE
Heart Disease (e.g., Angina or Chest Pain)	☐	☐	☐
History of Heart Attack or Heart Surgery	☐	☐	☐
Abnormal Electrocardiogram (ECG, EKG, Heart Tracing)	☐	☐	☐
High Blood Pressure	☐	☐	☐
High Cholesterol	☐	☐	☐
Blood Clots or Phlebitis	☐	☐	☐
Blood Disorders (e.g., Iron Deficiency)	☐	☐	☐
Breathing Disorders (e.g., Asthma, Bronchitis, Emphysema)	☐	☐	☐
Allergies	☐	☐	☐
Stroke	☐	☐	☐
Seizure	☐	☐	☐
Neurologic Disorder (Nerve, Spinal Cord or Brain Disorder)	☐	☐	☐
Significant Vision or Hearing Disorders	☐	☐	☐
Thyroid Disorder	☐	☐	☐
Diabetes or High Blood Sugar	☐	☐	☐
Cancer or Leukemia	☐	☐	☐
Auto-immune Diseases (e.g., HIV, AIDS, Lupus)	☐	☐	☐
Arthritis Other Than Osteoarthritis (e.g., Rheumatoid Arthritis)	☐	☐	☐
Musculoskeletal Disorder (Bone or Muscle Disorder)	☐	☐	☐
Liver or Digestive Disorder (e.g., stomach, intestine)	☐	☐	☐
Gastroesophageal Reflux Disease (GERD) or Frequent Heart Burn	☐	☐	☐
Do you smoke? If yes, how much? __cigarettes/day ___packs/week	☐	☐	☐

Please list any medications you are currently taking: ___________________________________
____
____

Health History Follow-up Questionnaire

Participant Number: ______________

Follow up questions to ask if the patient answered "yes" to any of the questions on the Demographic and Health History Questionnaire:

Have you injured either knee/hip?

1. Do you remember the diagnosis?
   - ☐ Yes If yes: What was your injury?
   - ☐ No
2. When did the injury occur?
   - ☐ < 1 year ago
   - ☐ 1-10 years
   - ☐ > 10 years
3. Was the injury sports related?
   - ☐ Yes
   - ☐ No

Have you ever had knee/hip surgery?

1. Do you remember what the surgery was for?
   - ☐ Yes If Yes: What was your surgery?
   - ☐ No
2. Was the surgery for osteoarthritis?
   - ☐ Yes
   - ☐ No
3. When was the surgery?
   - ☐ < 1 year ago
   - ☐ 1-10 years
   - ☐ > 10 years

Besides your knees/hips, have you been diagnosed with arthritis in any other joint in your body?

1. If yes, what joints?
   - ☐ Foot (R / L) ☐ Ankle (R / L) ☐ Knee (R / L) ☐ Hip (R / L)
   - ☐ Spine ☐ Shoulder (R / L) ☐ Elbow (R / L) ☐ Hand(R/L)
2. Was it osteoarthritis or another form of arthritis?
   - ☐ Osteoarthritis
   - ☐ Gout
   - ☐ Rheumatoid Arthritis
   - ☐ Other: ____

If you have ever received treatments for joint pain or arthritis, have you tired?

Over the counter drugs?

- ☐ Yes
- ☐ No

Prescription Medicines?

- ☐ Yes
- ☐ No

Corticosteroid Injections?

- ☐ Yes
- ☐ No

Hyaluronan/Hyaluronic Acid Injections?

- ☐ Yes
- ☐ No

Exercise or Rehab?

- ☐ Yes
- ☐ No

Herbal or Nutritional Supplements?

- ☐ Yes
- ☐ No

If you have ever played sports?

1. How long did you play the sports you listed?
   - ☐ ≤ 4 years
   - ☐ > 4 years
2. What was the highest level of competition?
   - ☐ Recreation
   - ☐ High School
   - ☐ College
   - ☐ Semi-Professional
   - ☐ Professional

Participant #: ______________

Pharmacological Use Assessment Questionnaire

Please mark if you have taken any of the following medicines or supplements for your arthritis or joint pain during the listed time periods. Please mark all that apply.

	Last 24hrs	2 weeks ago	2-4 wks ago	1-3mo ago	3+ mo ago
1. Anti-Inflammatory					
a. Ibuprofen (Motrin)	☐	☐	☐	☐	☐
b. Naproxen Sodium e.g., Naproxen, Naprosyn, Aleve	☐	☐	☐	☐	☐
c. Diflunisal (Dolobid)	☐	☐	☐	☐	☐
d. Ketoprofen (Orudis)	☐	☐	☐	☐	☐
e. Nabumetone (Relafen)	☐	☐	☐	☐	☐
f. Piroxicam (Feldene)	☐	☐	☐	☐	☐
g. Diclofenac (Voltaren)	☐	☐	☐	☐	☐
h. Indomethacin (Indocin)	☐	☐	☐	☐	☐
i. Tolmetin (Tolectin)	☐	☐	☐	☐	☐
j. Etodolac (Lodine)	☐	☐	☐	☐	☐
k. Ketorolac (Toradol)	☐	☐	☐	☐	☐
l. Oxaprozin (Daypro)	☐	☐	☐	☐	☐
m. Celecoxib (Celebrex)	☐	☐	☐	☐	☐
n. Aspirin e.g., Bayer, Acetylsalicylic Acid	☐	☐	☐	☐	☐
o. Other Anti-Inflammatory: ________________________________	☐	☐	☐	☐	☐
2. Herbal Remedy: ____	☐	☐	☐	☐	☐
3. Nutritional Supplement: _________________________________	☐	☐	☐	☐	☐
4. Injection: ____	☐	☐	☐	☐	☐
5. Tylenol (Acetaminophen)	☐	☐	☐	☐	☐
6. Other Medication for Pain: _______________________________	☐	☐	☐	☐	☐
7. Other Drug for Joints: ___________________________________	☐	☐	☐	☐	☐

<u>Pharmacological Use Follow-up Questionnaire</u>

Follow-up questions for each indicated drug:

Drug Name: __________

1. Do you know the dose?
   - ☐ Yes   If yes: What is/was the dose? _______mg _______times/day
   - ☐ No

2. Was the drug purchased over the counter or was it prescribed?
   - ☐ OTC
   - ☐ Rx

3. Was the drug taken as directed?
   - ☐ Yes
   - ☐ No

4. Was it taken consistently (as prescribed) for at least two weeks?
   - ☐ Yes
   - ☐ No

5. Who recommended it?
   - ☐ PCP
   - ☐ Ortho
   - ☐ Self Prescribed/OTC
   - ☐ Other: ___________

6. If you took more than one drug in a time period:

   Did you take these medicines in the same day?
   - ☐ Yes
   - ☐ No

   If yes, did you take them at the same time of day?
   - ☐ Yes
   - ☐ No

7. If you stopped using the drug, why did you stop using the drug?
   - ☐ GI Issues / Adverse Interactions
   - ☐ Didn't work
   - ☐ Other: ________________________

8. During the last two weeks, have you used an additional drug during the day for unexpected symptoms?
   - ☐ Yes
   - ☐ No

   If yes, what was it? ________________________
